# Let’s talk about pain: A mixed methods exploration of patients’ pain knowledge and preferred ways to introduce the biopsychosocial model of pain

**DOI:** 10.1080/24740527.2026.2650304

**Published:** 2026-05-11

**Authors:** Cynthia J. Thomson, Katherine N. Holden, Luisa V. Giles, Leanne Cianfrini

**Affiliations:** a Faculty of Health Sciences, School of Kinesiology, University of the Fraser Valley, Chilliwack, British Columbia, Canada; b Actum Health, Surrey, British Columbia, Canada

**Keywords:** Biopsychosocial model, focus group, pain education, chronic pain, persistent pain, patient-oriented, knowledge translation

## Abstract

**Background:**

Clinical guidelines recommend treating chronic pain using a biopsychosocial (BPS) model, yet the biomedical model remains dominant. It is known that pain is complex and influenced by many factors but, to our knowledge, patient-centered ways to introduce the BPS model have not been explored.

**Aims:**

Our primary aim was to explore patient-centered ways to introduce the BPS model of pain; second to this, we evaluated any changes in pain knowledge from baseline to follow-up.

**Methods:**

We facilitated focus groups with people (*n* = 21) living with chronic pain. We employed blended reflexive and codebook thematic analysis to identify themes and subthemes and measured knowledge change (pre–post) using a paired *t-*test.

**Results:**

We developed themes that pertained to knowledge of, and barriers and facilitators to, sharing the BPS model of pain. Participants reported a lack of knowledge sharing by providers, and the gap was supported by low accuracy on a pain physiology questionnaire. We interpreted barriers to knowledge sharing as bidirectional: with systemic barriers affecting knowledge delivery (i.e., time, biomedical bias) and person-level barriers affecting knowledge receptivity (i.e., fear of stigma, history of diagnoses). An approach involving meaningful validation, individualized tailoring, multiple modalities (i.e., posters, handouts, videos), and knowledge sharing by all providers earlier in the pain journey may serve as facilitators to support a BPS-focused dialogue.

**Conclusions:**

Participants expressed a need for BPS model knowledge sharing. A patient-centered approach will prioritize validation, individualization, and the value of personal narratives for illustrating the brain’s role in the pain experience.

## RÉSUMÉ

**Contexte :** Les lignes directrices cliniques recommandent le traitement de la douleur chronique à l’aide d’un modèle biopsychosocial (BPS), mais le modèle biomédical demeure prédominant. Bien qu’il soit reconnu que la douleur est complexe et influencée par de nombreux facteurs, à notre connaissance, les façons centrées sur la personne d’introduire le modèle BPS n’ont pas encore été explorées.

**Objectifs :** L’objectif principal de cette étude était d’explorer des façons centrées sur la personne d’introduire le modèle BPS de la douleur. À titre d’objectif secondaire, nous avons évalué les changements dans les connaissances sur la douleur entre l’état initial et le suivi.

**Méthodes :** Nous avons animé des groupes de discussion auprès de personnes (n = 21) vivant avec une douleur chronique. Nous avons réalisé une analyse thématique combinant des approches réflexives et fondées sur un cadre de codage (« codebook ») afin d’identifier des thèmes et des sous-thèmes. Nous avons ensuite mesuré les changements dans les connaissances (pré–post) à l’aide d’un test *t* apparié.

**Résultats :** Nous avons dégagé des thèmes portant sur les connaissances relatives au modèle BPS de la douleur, ainsi que sur les obstacles au partage des connaissances et les facteurs qui le facilitent. Les participant(e)s ont signalé un manque de transmission des connaissances de la part des professionnel(le)s de la santé, lacune corroborée par un faible taux de réponses exactes à un questionnaire sur la physiologie de la douleur. Nous avons interprété les obstacles au partage des connaissances comme bidirectionnels : des obstacles systémiques influençant la diffusion des connaissances (p. ex., contraintes de temps, biais biomédical) et des obstacles à l’échelle individuelle influençant la réceptivité aux connaissances (p. ex., crainte de la stigmatisation, antécédents diagnostiques). Une approche misant sur une validation significative, une adaptation individualisée, le recours à de multiples modalités (p. ex., affiches, documents d’information, vidéos), ainsi que le partage des connaissances par l’ensemble des professionnel(le)s dès les premières étapes du parcours de douleur, pourrait faciliter un dialogue axé sur le modèle BPS.

**Conclusions :** Les participant(e)s ont exprimé un besoin de partage des connaissances relatives au modèle BPS. Une approche centrée sur la personne devrait privilégier la validation, l’individualisation et la valeur des récits personnels pour illustrer le rôle du cerveau dans l’expérience de la douleur.

## Introduction

Chronic pain is a complex phenomenon that is described as “an unpleasant sensory and emotional experience associated with, or resembling that associated with, actual or potential tissue damage. (p. 1)”^1^ The biopsychosocial (BPS) model recognizes the complex interplay between multiple factors, including biological, psychological, and sociological dimensions, in the manifestation of both acute and chronic pain.^2^ Despite the dominance of the BPS model for the conceptualization of pain in the literature, its uptake by health care professionals (HCPs) is suboptimal.^3–6^ Formal training in pain science for HCPs is lacking,^7^ and there remains a gross overemphasis on treating pain through a biomedical lens.^4,6^ Compounding the problem are pervasive public views of chronic pain, which are still largely based on a reductionist model linking pain to tissue damage.^4,5,8^

The lack of uptake combined with public misconceptions has likely contributed to an overuse of treatments focused on the sensory or physical aspects of pain (e.g., manual therapies, pharmaceuticals, passive physiotherapy modalities). Biomedical treatments place less emphasis on treating the psychological, emotional experience of chronic pain^6,9^ and neglect to consider and address the complex social factors associated with pain.^10^ HCPs cite patient expectations for biomedical interventions as a barrier to recommending BPS-focused treatments.^11^ Given that treatment recommendations are often guided by client choice, even a BPS-centered professional may emphasize biomedical treatments and neglect to offer other options to appease their patients.^11^ A range of psychologically based treatments have been shown to be effective in treating chronic pain.^9,12,13^ Pain education has been shown to be a standalone effective method to reduce pain and mental health outcomes,^14–17^ with enhanced outcomes when learning objectives are achieved^18^ and when couples (person living with pain and their partner) are both educated about pain.^19^ Recently, multimodal treatments that included pain education combined with sensorimotor retraining or pain reprocessing resulted in moderate to large effect sizes in patients with chronic low back pain (LBP) and heterogeneous pain conditions.^20–22^ Interventions aimed at ameliorating social factors contributing to pain, including social prescribing and peer support, have also shown small effects on pain outcomes, though more research in this area is warranted.^23,24^ Many evidence-based and freely available resources exist online to provide education about the science of pain (e.g., “how pain works”),^18^ but if a patient believes that their pain is purely physical, they may not understand why a clinician is suggesting a website for education, a peer support group, or a psychologically focused treatment rather than a physical treatment modality. For example, Young et al.,^25^ recounted a patient’s confusion: “What are you giving me antidepressants for? I’m not depressed, my back hurts—that’s why I’m depressed and you people are depressing me because you’re not listening.”^(p207)^

Lack of understanding and conflicting health beliefs can decrease patient compliance for a treatment,^26^ thereby impacting the success of prescribing an educational resource without supportive rationale. The pain science educational resources are plenty, but there exists a gap regarding how to first introduce the concept of the BPS model of pain to patients. A barrier cited by clinicians trying to integrate the BPS model into practice is the lack of a “sound bite” that is comprehensible to patients to accurately describe the neurophysiology of pain.^11^ Introducing the BPS model to patients can be challenging because there is a common misunderstanding that “pain in the brain” equates to “pain is in the head” (or imagined); additionally, some patients are resistant (“don’t want it”) or lack trust (“don’t buy it”).^27^ Pain is not imagined: all pain is real, and it is commonly life-altering and debilitating. Few studies have explored ways to introduce the BPS model of pain from a patient perspective.^28,29^

The primary objective of this mixed method, patient-partnered study was to explore preferred ways to introduce the BPS model to people living with pain using qualitative reflexive thematic analysis of focus group data.^30,31^ Our secondary objective was to quantitatively explore participant knowledge change by comparing pre– and post–focus group scores from a pain neurophysiology questionnaire. BPS-focused treatments may only be considered by patients if clinicians can sensitively communicate the foundational fact that pain is perceived in the brain. To support meaningful knowledge creation and guided by the strategy for patient-oriented research,^32^ we engaged patient partners in all stages of the research, from study design and focus group script development to analysis and knowledge translation.

## Materials and methods

### Study design

We employed mixed methods to explore (1) preferred ways to introduce the BPS model of pain to individuals living with chronic pain (qualitative, focus groups) and (2) knowledge change following a brief introductory lesson and focus group discussions (quantitative, survey based). Our qualitative methods were informed by a reflexive thematic analysis framework and adopted a relativist-constructivist approach, such that multiple realities exist and through focus group exchanges we can generate a contextual understanding of the topic. We provide further details in the analysis section. Participants provided informed consent through the online survey (at baseline), and the University of the Fraser Valley Human Research Ethics Board in British Columbia, Canada, approved procedures (HREB No. 101389). All procedures are in accordance with the Declaration of Helsinki. Participants received a gift card ($15) as an honorarium for participation. Patient partners received a $500 honorarium for their participation throughout the research process. Further details about patient partners and team collaboration are found in the supplementary file.

### Participants

Participants responded to recruitment posts on social media, campus and health center posters, and a patient recruitment platform called Reach BC (www.reachbc.ca). Interested participants (*n* = 27) were prescreened for eligibility via e-mail and eligibility was confirmed during the baseline survey. Eligibility criteria required participants to be the legal age of majority in British Columbia (19 years of age), have chronic pain for at least 6 months, and to experience pain on at least half of these days in the last 6 months. Exclusions included psychotic disorders, problematic substance abuse, and/or diagnosis of specific inflammatory disorder(s) that commonly include multiple symptoms beyond pain (e.g., rheumatoid arthritis, lupus). Of the prescreened participants, six were not included in the study due to the following: did not consent (1), failed survey screening (1), incomplete survey (1), and unable to attend focus group (3).

### Procedures

In fall 2023, participants completed baseline surveys (online through surveymonkey.com) that inquired about demographic characteristics, pain history, pain severity, and interference with daily living (Brief Pain Inventory–Short Form [BPI-SF]; permission for use obtained through MD Anderson, www.mdanderson.org),^33^ pain intensity and interference (Patient-Reported Outcome Measurement Information System [PROMIS] short forms 3a and 8a),^34^ along with an assessment of knowledge about the neurophysiology of pain (described in Measures). Though redundant, both pain assessment tools are commonly used in clinical and research settings. Eligible participants were assigned a focus group based on availability and format preference (online versus in person). Prior to the focus group, participants viewed a 15-min lesson (details below) to introduce them to the BPS model and provide them with facts about pain. Each participant then attended a 90-min semistructured focus group either virtually or in person. Within 2 days of the focus group, participants received a link to complete a post–focus group survey that included one questionnaire repeated from the baseline survey (the Neurophysiology of Pain Questionnaire), along with additional measures to describe the psychological characteristics of the sample. These included the following: beliefs about pain using the Pain Catastrophizing Scale (PCS^35^; permission for use obtained through Mapi Research Trust, http://eprovide.mapi-trust.org), screening tools for anxiety (Generalized Anxiety Disorder 7-item scale [GAD-7])^36^ and depression (Personal Health Questionnaire [PHQ-8],^37^ and a quality of life measure (the Short Form Quality of Life [SF-12]).^38^ The aforementioned psychological constructs are associated with chronic pain and are commonly used to characterize samples.^39^ All tools used to describe sample characteristics demonstrated high internal reliabilities based on Cronbach alphas in the current sample (⍺ = 0.91–0.96).

#### Pre–focus group lesson

To ensure all participants entered the focus group with basic knowledge about the current model of pain that would be the focus of the discussion, participants completed a brief, self-paced online presentation (see Supplementary Material) that provided an overview of the BPS model along with facts about chronic pain. The lesson included two evidence-informed, 5-min videos about chronic pain, both developed by pain scientists/content experts (Tame the Beast^40^: https://www.tamethebeast.org/; Understanding pain in less than 5 minutes^41^: https://www.youtube.com/watch?v=C_3phB93rvI). We confirmed lesson completion via a link at the end of the presentation.

#### Focus group

We aimed to include five to eight participants per focus group,^42^ each lasting 90 min and led by a trained facilitator who is also a practicing pain psychologist. One patient partner and/or researcher was also in attendance. Informed by participant preference, three focus groups occurred online via Zoom^43^ with participant videos turned on and one group occurred in person at our university campus. Focus groups were recorded using the Zoom audio recording system for online meetings and a laptop with an external microphone for the in-person session. Our study team (including patient partners) drafted the scripts for the focus groups, consisting of a semistructured approach with guiding questions and free-form follow-up questions determined by the facilitator (see supplementary file). We selected focus groups as our method for data collection (in lieu of interviews) to enable opportunities to build upon other viewpoints (“piggybacking”), which we felt may promote candid responses.^44^

### Quantitative measure

#### Revised Neurophysiology of Pain Questionnaire

The Revised Neurophysiology of Pain Questionnaire (NPQ-R) was designed to measure a person’s knowledge of the biology of pain.^45^ It includes 13 statements about the neuroscience of pain with three response options: true, false, or undecided. Points are awarded for correct responses and summed. Scores range from 0 to 13, with higher scores indicating greater knowledge of the neuroscience of pain. The NPQ-R demonstrated moderate reliability in the current sample at baseline (⍺ = 0.64).

### Analysis

#### Qualitative analysis

A research assistant (K.N.H.) transcribed the focus group discussions verbatim using Microsoft Word 365 speech-to-text transcription software while overseeing and editing as needed. We conducted member reflections with all participants to ensure the text represented the participants’ beliefs and provided opportunities for reflexive elaboration following the focus group.^46^ Three researchers (C.J.T., L.V.G., L.C.) and a research assistant (K.N.H.) independently coded the transcripts using the cloud-based software Delve.^47^ The method we employed was reflexive inductive thematic analysis, informed by Braun and Clarke,^30,31,48^ whereby we practiced reflexivity throughout the study (details on our positionality described later). Our method involved transcript familiarization through multiple readings followed by coding and multiple iterations of theme development and refinement.^30,31^ Inductive thematic analysis is a data-driven process where themes are derived from the data, rather than using theory to guide selections. Though we primarily adopted a reflexive approach, during analysis this evolved into a blended approach that included both inductive reflexive and codebook thematic analysis.^31,48^ We did not have a preestablished codebook, but we recognize that our coding was influenced by a pragmatic consideration to meet the information needs relevant to our research question and future knowledge mobilization applications (i.e., identify a knowledge gap, along with a “how to” introduce the model).

During the first round of coding, the coders independently labeled raw quotes, grouped them by codes/categories, and then organized and refined the labeling of the codes. The researchers met to discuss their individual results and explored overlapping or contrasting codes and categories to develop candidate themes. We then met with patient partners to collaboratively discuss and refine themes and subthemes. The researchers then revisited their coded transcripts (with the overarching themes in mind) and actively reflected whether the themes represented the data and noted discrepancies. Finally, researchers regrouped, edited, and finalized themes and developed and/or collapsed subthemes when relevant. Through collaboration, the team selected quotes that best represented the data. Our themes have both semantic and latent meaning; for example, some themes that emerged directly from questions posed by the facilitator (informed by our research objectives) are semantic in nature, but we also abstracted from underlying threads in the data to craft latent themes. When presenting results, the authors edited the verbatim text to intelligent verbatim transcript by removing filler and repeated words and identified participants by number P1 through P21 and focus group FG1 through FG4. We provide descriptors for pain conditions, gender, and age in Table 1.

**Table 1.** Individual participant demographics.

Study ID	Age	Sex	Condition or location of pain	Pain duration (years)	Pain severity
P1	70	Female	OA, LBP	14	8
P2	60	Female	OA, DD, widespread pain	44	7
P3	63	Male	LBP	3	7
P4	51	Female	No diagnosis, localized MSK (arm)	<1	4
P5	74	Female	OA, DD, knee	7	6
P6	62	Female	No diagnosis, widespread	13	6
P7	61	Male	LBP, DD	4	4
P8	66	Female	No diagnosis, LBP	51	3
P9	59	Female	No diagnosis, LBP/hip	16	3
P10	65	Female	No diagnosis, localized MSK (shoulder)	20	1
P11	32	Female	No diagnosis, back pain	2	7
P12	74	Female	Scoliosis	3	6
P13	65	Female	OA (shoulder)	10	4
P14	58	Female	OA, DD, FM	22	5
P15	55	Female	No diagnosis, localized MSK (shoulder)	1	2
P16	32	Female	Tendonitis (shoulder)	4	4
P17	78	Male	No diagnosis, LBP	8	5
P18	43	Female	No diagnosis, localized MSK (shoulder)	5	3
P19	51	Female	Long covid (facial/head)	3	5
P20	74	Female	OA, FM	30	6
P21	56	Female	OA, EDS, scoliosis, widespread	43	5

Pain duration has been rounded to the nearest year. Pain severity is based on the BPI item “pain on average.”

##### Reflexivity

We acknowledge that researcher background influences data collection, analysis, and interpretation. Two of the authors have lived experience with chronic pain (CJT, LVG) and with treating their own pain using a BPS approach. is a practicing pain psychologist (LC) who employs the BPS approach to caring for patients, and she served as the facilitator of the focus groups. lives with dysautonomia (KNH) and employs the BPS approach to help manage her symptoms. Our respective positionalities (including those of our partners) influenced the development of the facilitator script and survey items and the inclusion of the knowledge assessment tool. Our experiences highlighted a lack of BPS model knowledge sharing, either personally or professionally; therefore, we anticipated lack of knowledge and/or misinterpretations of the BPS model of pain. This assumption informed the inclusion of a pre–focus group lesson, a pre–post analysis, and exploring participant reactions to the shared materials (we suggested they take note of any reactions to the lesson).

#### Quantitative analysis

To describe the sample, total and average scores for questionnaires are presented using means and standard deviations and diagnostic categories are noted using frequency data. To assess any changes in knowledge about the physiology of pain, we compared baseline NPQ-R scores to follow-up scores using paired *t*-tests. Significance was set at *p* < 0.05.

##### Sample size

Our target sample size was between 20 to 30 participants or four to eight focus groups.^49^ We aimed to recruit enough participants to enable representation of diverse viewpoints (e.g., age, gender, education) and pain experiences (e.g., duration, condition, knowledge base). Given that assessing knowledge change through statistical analysis was a secondary objective, we did not carry out a priori power calculation for changes in NPQ-R.

## Results

Twenty-one participants from British Columbia (18 women, 3 men; *M*_age_ = 59.5 [SD = 12.6] years) with chronic pain (mean duration 14.4 [SD = 15.3] years) participated in this study. Summary demographic information, pain characteristics, and additional features that are commonly associated with chronic pain are shown in Table 2. Our sample was well educated (all reporting a diploma or higher) and largely represented a middle household income with variation across the levels within the middle bracket. The sample reported diverse employment status, with a subset of retired (43%) and employed (33%) participants and some representation of those unable to work (14%). Not all participants had a formal diagnosis for a pain condition (no diagnosis: *n =* 9, 43%). Of the participants with a formal diagnosis (*n* = 12, 57%), osteoarthritis (OA) was the most common condition reported (*n* = 7, 33%). Our sample also included participants with fibromyalgia (FM, *n =* 2), long COVID–associated pain (*n =* 1), reports of diagnosed degenerative discs (DD, *n =* 4), and scoliosis (*n =* 2; (frequencies are not mutually exclusive). Those noting DD or “bulged disc” provided anatomical locations, and we have noted these as LBP (*n* = 6) in individual participant descriptors (Table 1).

**Table 2.** Participant demographics.

Characteristic	*n* = 21
Demographic features	
Education, *n* (%)	
High school or less	0
Trade or diploma	8 (38.1)
Bachelor’s degree or higher	13 (61.9)
Household income, *n* (%)	
<$34,999	3 (14.3)
$35,000–$49,999	2 (9.5)
$50,000–$74,999	5 (23.8)
$74,999–$99,999	6 (28.6)
>$100,0000	3 (14.3)
Did not disclose	2 (9.5)
Employment status, *n* (%)	
Unable to work/on disability	3 (14.3)
Employed (full and part time)	7 (33.3)
Other (student, homemaker)	1 (4.8)
Retired	9 (42.9)
Did not disclose	1 (4.8)
Relationship status, *n* (%)	
Single	4 (19.1)
Married/common law	12 (57.1)
Separated/divorced	5 (23.8)
Clinical features, mean (SD)	
BPI mean severity (0–10)	4.5 (1.7)
BPI mean interference (0–10)	4.5 (2.5)
PROMIS intensity sum (3–15)	8.9 (1.7)
PROMIS interference (*t-*scores: 0–100)	61.7 (6.5)
Psychological features, mean (SD)	
Pain catastrophizing (PCS) (0–52)	14.65 (10.0)
Depression (PHQ-8) (0–24)	7.55 (5.1)
Anxiety (GAD-7) (0–21)	7.2 (5.7)
SF-12 Quality of Life, Physical score (0–100)	29.8 (10.8)
SF-12 Quality of Life, Mental score (0–100)	50.3 (9.4)

The mode values were imputed for each of the single missing data points from the PCS, PHQ-8, and GAD-7 (all in different individuals).

Overall, our sample reported mild anxiety, with most (*n =* 11, 55%) participants meeting the criteria for mild anxiety (0–5 points). Anxiety scores were not significantly different from a large study that combined multiple chronic pain samples.^50^ The pattern for depressive scores was similar, with an average score below the threshold (>10 points) for clinical depression comprising 65% of the sample, though 35% (*n* = 7) of participants met the criteria for major depression,^37^ a prevalence that is similar to a large representative American sample with chronic pain affecting daily living.^51^ The average score for pain catastrophizing in our sample (14.7 [SD = 10]) is considered not clinically relevant (cutoff score ≥30). Participants reported low scores (<30) for the physical component score for quality of life as measured by the SF-12 falling well below the 50th percentile. For the mental component score (SF-12, quality of life), participants reported scores on par with a general population median of 50.

### Quantitative analysis

We compared pain knowledge between baseline and follow-up using a paired *t-*test. One participant did not respond to follow-up surveys, leaving *n* = 20. There was a large effect size (*d* = 1.96), depicting an increase in knowledge of the neurophysiology of pain (*t*[19] = 4.68, *p* < .001, 95% confidence interval 1.13, 2.97). Participant knowledge increased from an average score of 5.35 (SD = 2.25) to 7.40 (SD = 1.54) following the combination of the 15-min lesson and 90-min focus group.

### Thematic analysis

Through blended reflexive and codebook thematic analysis, we gained insight into client preferences surrounding a sensitive introduction to the BPS model of pain. We constructed six themes:

1. We never heard it from them [HCPs]: Participants have some knowledge of the BPS model, largely self-taught.

2. Systemic barriers affect knowledge delivery.

3. Patient beliefs affect knowledge receptivity.

4. Validate my pain!

5. Introducing the BPS model: Who, what, when, how, and why?

6. Storytelling: Individualized evidence for the brain’s role in the manifestation of chronic pain.

#### Theme 1: We never heard it from them [HCPs]: Participants have some knowledge of the BPS model, largely self-taught

Most participants reported learning about certain aspects of the BPS model of pain during their pain journey, though not from their HCPs, and the model was not well understood. Participants largely reported learning about the BPS model on their own through books, videos, and online courses. For example, “I haven’t been told this by any treatment provider. But I have read a book” (P10) and “I read a book, too” (P8). Similarly, P18, with localized musculoskeletal (MSK) pain stated, “I stumbled upon the information in researching what I was experiencing, but I never was provided any guidance or information on it from the 15 medical professionals that I saw.” We noted that three participants reported learning about it from health care providers (i.e., multidisciplinary pain clinic, pain program at an athletic center). P14 with multiple pain diagnoses described her reaction to her first introduction to the model: “It’s information that I had heard before, but it was when I was first injured and the way it was presented to me, I had an adverse reaction to it and rejected the idea.”

Despite most participants reporting some familiarity with the BPS model of pain, there was still confusion and/or dissent surrounding the concept that tissue damage is not correlated with pain experience (see Theme 3: Patient beliefs affect knowledge receptivity). Furthermore, several participants reported learning something new through the introductory lesson; for example: “For me that was totally a new concept: that pain was not in my head, but it was in my brain” (P8).

#### Theme 2: Systemic barriers affect knowledge delivery

Participants spoke of barriers related to our (Canadian) health care system and to societal views that impact knowledge sharing about the BPS model. We created three subthemes related to barriers to knowledge delivery: (1) Not enough time, (2) Bias toward the biomedical [model], and (3) Do they [HCPs] know? Perceived lack of HCP knowledge.

##### Not enough time

Several participants noted that our health care system prevents practitioners from taking enough time for “understanding who your patient truly is” (P4) or to adequately provide support. A 10- to 15-min appointment is considered “too truncated” (P11) to introduce the BPS model or suggest alternative pain treatments.

When you only have a time slot of 10-minutes and someone who may see you once a year, because I don’t go to the doctor very much. … There was no understanding of who I am, my point of reference for anything. In a 10-minute time slot, how well are they going to be able to address you, let alone understand who you are? (P4)

The limited time common to medical appointments in our system aligns with theme 5 (subtheme related to “who” should introduce the BPS model), suggesting that multiple, diverse practitioners need to be involved in sharing the information. A participant described the brief interactions with a surgeon, compared to the regular follow-up interaction with physiotherapists; this was echoed by several participants: “Just to build on that again, too, my experience with surgeries has been—I may see the orthopedic surgeon three times. And after that, [the patient] is handed over to the physiotherapist” (P3). Continuity of care and limited time came up several times throughout the focus group as an obstacle to sharing BPS knowledge with people living with pain.

*Bias toward the biomedical [model]*. Participants described the dominance of biomedical diagnostics and treatments and lack of BPS-informed options. These participant accounts provided the basis for the idea that a biomedical bias in providers hinders information sharing about the BPS model. They spoke of physicians not knowing what to do with them or “they kind of [have] written you off at some point” (P10) or “wash their hands” (P18). They described that physicians had nothing to offer because they either could not find a biomedical cause for the pain or the pain had “gone past the normal [healing] timeline” (P10). For example, P13 with OA said, “[The doctor said], ‘Well, there’s nothing we can do.’ And I thought, ‘Can’t you even refer me to a peer support group or something about this issue?’” Another participant (P5) described sharing information about the BPS model following an online pain course with her doctor: “Well, I talked to my orthopedic surgeon about it and he pretty well dismissed it.” Taken together, the comments provide support for a latent subtheme that represents a bias toward treating the biomedical aspects of pain as a systemic barrier to BPS model introduction, thus leaving patients and providers without strategies once biomedical routes have been exhausted.

In my experience, most doctors come at it like you said, from the biomedical aspect, and then once they can’t identify that, then they’re no longer necessarily interested in the case anymore. (P18)

Multiple participants reported that doctors offered prescriptions and had no alternative suggestions. “No, that’s not why I’m here. I don’t actually want a prescription. I want something else” (P14). Several participants reported that they did not visit their doctor with an intention to obtain a prescription and were seeking nonpharmacological treatment options. Such consults may have been lost opportunities to introduce and start a dialogue about BPS factors that are known to influence pain, alongside redirection toward widely available psychosocial treatment options.

##### Do they [HCPs] know? Perceived lack of HCP knowledge

The final systemic barrier to information delivery centers on provider knowledge (or lack thereof). Participants questioned whether their HCPs have knowledge of the BPS model of pain: “I don’t know that they have any training, … right?” (P3). Our patient partner (FG1, back pain) acknowledged that HCPs would need to actively seek out and invest in training to stay current, “and to be able to have the knowledge that was shared with us on these films and through the course, I think physicians themselves, even though it’s a time investment, it’s an investment.” Participants were not aware that clinical guidelines recommend a BPS-informed approach for chronic pain management.

Are doctors aware of all these options that they can present? I mean, do they know about the support groups, and if they do, they should be just offering, putting it out there helping, but maybe a lot of them aren’t even aware of the resources. (P9)

Given that most of our participants had never been introduced to the BPS model by a health care provider, many made assumptions that providers must not be aware of the model, “It seems like all these doctors that you guys mentioned [referring to other participants’ doctor experiences], it doesn’t sound like they are aware that it’s a possibility, so they don’t mention it?” (P8). It was assumed that if HCPs were aware, they would have shared ideas regarding the BPS model of pain.

#### Theme 3: Patient beliefs affect knowledge receptivity

We identified two subthemes that may influence the latent theme that client beliefs affect openness to learning about the BPS model. Several participants reported confusion regarding the biopsychosocial contributions to pain (Does the BPS still apply to me?), and participants described adverse reactions when attributions to psychological influences were implied (I’m not crazy!).

*But I Have Tissue Damage, Does the BPS Still Apply to Me?* There was some confusion from participants regarding the relative contributions and bidirectional nature of relationships within the BPS model. Though many participants reported having some knowledge of the model (theme 1), several participants were unsure whether the model applied to them, because they have “degenerative issues” or “injuries,” and therefore the role of the brain in perception seemed less applicable to them. For example, a participant (P1) with OA and LBP expressed her confusion with the brain’s role in pain: “When I hear you say your brain could be relearned. But mine’s [pain] in my bone. How do you tell your bones not to hurt?” Another participant (P19) with long COVID whose pain is localized within her head (and brain) struggled with thinking about the brain’s role in pain perception, stating that “it hurts up here where my brain is … so we’re talking about the brain being at play and it’s like, well, but that’s where all my pain is.”

One pain fact that we presented in the pre–focus group lesson, “pain is not an accurate reflection of tissue damage,” appeared to be a sticking point for many participants, and some of them questioned the relative contributions of the psychosocial influences of pain. “How can we determine at what point it isn’t anything that we should continue to address by a physio[therapist] or by something along those lines, and when it is more from the psychology?” (P11). The lack of direct correlation between tissue damage and pain intensity was especially difficult for participants who had received formal diagnoses from physicians (e.g., history of injuries, OA, Ehlers-Danlos syndrome [EDS]).

So [I am] a little conflicted, lots of sports injuries, multiple surgeries, recovered from the pain of ACL tears, things like that. So, one injury I had about five years ago continues to plague me and it leads to muscle cramping and spasm so that’s got me wondering, what about social phenomena? […] If it’s muscle cramps, it’s my entire back that will do it and I can’t breathe, so is that the brain triggering that or […] the muscles cramping?

The same participant also said,

My physio was reminding me, “Don’t forget you have a herniated disc and we’re trying to get control of that.” So the pain I have when I can’t get dressed in the morning, my gut feeling is that’s not biopsychosocial—that’s a herniated disc switching on there. But if it’s not that, if it’s this, then how do I get my head around that? (P3)

Further affecting the receptivity to the message, several participants wanted to quantify the psychosocial contribution and verify applicability; for example, does this apply to my condition and what percentage of it is psychological or sociological for me? “It [the lesson] makes sense to me, so it was just quite complex, so I’m curious … how much do each play a role?” (P19). Another participant (P21) with multiple diagnoses wondered how degenerative conditions fit within the BPS model,

Where it [referring to the lesson] says, the brain is faulty for any chronic pain that lasts longer than six months—and I’m wondering how degenerative issues fit into that, like degenerative discs and osteoarthritis and Ehlers-Danlos syndrome, […] ’cause that’s the kind of thing that is not going to heal. Ever. Right? It’s just going to keep getting worse. So how does that fit in with this model?

There may be a tendency for those with formal diagnoses to be less open to the BPS model or to assume that the model does not apply to them. Prior diagnoses related to structural abnormalities are a potential barrier to knowledge receptivity. It will be important to emphasize that the BPS model of pain is relevant to all kinds of pain, whether acute or chronic, regardless of the condition, given that all pain is perceived in the brain.

##### I’m not crazy! Fear of pain being attributed to psychological influences

Openness to the BPS model, particularly the psychosocial elements, may be influenced by stigma related to mental health and lack of validation from HCPs. Participants recommended caution around implying that a client has mental health issues. A participant (P20) with FM and OA stated: “If you could figure out some way to call it other than mental health. Because for a lot of people you say mental health, ‘Well, I’m not crazy.’” Another participant (P13) noted her preference for describing the experience of pain in the “brain”: “I would rather somebody used the word ‘brain’ rather than ‘head,’ cause head kind of implies psychological, and [chuckles] maybe, you’re just dreaming it up.” Similarly, a participant (P21) noted the perceived conflation of psychological factors and invalidation that can occur: “A lot of the times when this information is presented, it’s all in your head, or it’s a psychological thing, or anxiety, it’s not presented in a healthy way.” This was echoed by another participant (P19) with long COVID:

When you’re communicating this [the model] to people, not saying or presenting it like, “Oh, you should go see a psychologist,” because a lot of times it’s like you’re a little bit cuckoo [chuckles], instead of, “You should look at your thoughts and emotions, things that might reduce the stress.” Because- a lot of times it was, “Have you seen a psychiatrist?”

Consistently, participants expressed a worry about the implications of discussing psychological factors contributing to pain, because psychological attributions may be perceived as invalidating. The same participant (P19) also cautioned about the potential for perceived blame, “You need to—you need to get a grip on your thoughts and emotions because you’re to blame.” Framing pain as a complex integration of messages perceived by the brain rather than attempting an oversimplification to one pathway (e.g., anxiety, stress) may reduce stigma and perceived blame and improve receptivity to the BPS model of pain.

#### Theme 4: Validate my pain!

Theme 4 was the most pervasive latent theme that we drew from participants’ dialogue. Participants consistently reported experiencing a lack of validation and described examples of dismissal and being made to feel that the provider believes the pain “is in [their] head” (P2, P6, P19, P21).

I have discovered that until my most recent doctor, the majority of doctors are not comfortable with the idea of chronic pain. And I’ve had everything from dismissal to accusations that I’m trying to get stronger drugs [chuckles]. (P14)

Examples of dismissal included not being listened to when concerns were brought forward, practitioners not taking the time to understand how pain affects a person’s everyday tasks, and HCPs telling them “to live with it” (P5) because it (most were referring to OA) is part of getting older.

I thought it was great! [referring to lesson/videos]. If I can figure out a way to get my brain to shut off some of the pain I feel [chuckles] that would be great. I’m all for it. [Be]cause basically I’ve been told that it’s just, “You’re aging, and these things happen, and things break down, and not much we can do for you, right?”(P7)

Further supporting a general lack of validation, many participants described situations where they felt their experiences were not believed or were imagined or it was implied by a practitioner that what they were describing was “in your head.”

I find you don’t want to say a lot because a lot of times the doctors don’t believe you or they say that you’re hallucinating or it’s in your head, so you don’t even want to say what you’re really feeling. So you kind of just be quiet. I don’t think a lot of them understand pain, like the way that we have it. (P2)

Participants also described a lack of validation from the perspective of not being heard and/or not taking the time to ask about the impact of pain on daily life. “They don’t see how it impacts you every single day, so then you just come and say I’m in pain all the time, but they don’t then ask how does that actually impact your day-to-day life” (P11). Most participants in our focus groups described exchanges where their pain experiences were not validated or understood. A sensitive introduction of the BPS model to patients requires active, empathetic listening; support for holistic management beyond pharmaceuticals; and an understanding that all pain is real and hugely impacts a person’s life.

#### Theme 5: Introducing the BPS model: Who, what, when, how, and why?

Theme 5 is a semantic theme that relates directly to scripted research questions and to the primary objective of the study. We identified subthemes (who, what, when, how, and why?) regarding various contextual factors related to BPS introduction. Supporting quotes for the subthemes described next are provided in Table 3 and numbered Q1 through Q17.

**Table 3.** Patient-centered ways to introduce the BPS model of pain (Theme 5: Who, what, when, how, and why?).

Subtheme	Exemplar quote		
Who? Everyone [all HCPs] should be sharing the BPS model	I feel that multiple practitioners should be sharing it because it involves multiple modalities. It’s not just your GP, and it’s not just your physiotherapist. […] It’s all those parts combined.	P6	Q1
	I haven’t actually spoken to my GP about some of the things that have become chronic.	P15	Q2
	If there was, like, a campaign or just promotional materials that were put in a doctor’s offices, […] people could absorb the information.	P18	Q3
What? Multiple tools can be used to share the knowledge	And also the posters on doctor’s offices are the best. I remember going into a doctor’s office […] and there was a sleep apnea poster on the wall, and when the doctor came in, I said, “I think I have that.” And got tested and, sure enough, those things really work.	P13	Q4
	I was sitting there waiting for my GP, the last time I was learning all about kidneys because they had this really interesting poster up on the wall, right? […] I’m learning that stuff, absorbing it, even if it’s not relevant to me at that moment. […] Next time, five years later, when something’s happening, I have that knowledge basis already.	P18	Q5
	This [referring to pharmacy handouts] allows me to come home and then further research and then I can kind of investigate further myself. So, I find that helpful.	P16	Q6
When? HCP should introduce the BPS model earlier, and not as a last resort!	If it’s introduced early on, but intentional like, this is going to be part of the treatment plan, versus as a last resort and they can’t figure out why you’re still having pain, […] [be]cause it feels more dismissive.	P11	Q7
	Maybe at the three-month mark when you’ve tried a couple of things, because at first, if they told me that [referring to BPS model], I would think you’re not listening to me and you’re just shoving me off onto the stupid program so you don’t have to deal with me.	P2	Q8
How? Work with me as a unique individual	For me, time is really important. If any practitioner or doctor or physiotherapist is willing to take the time to unravel it all, rather than making me feel like it’s a cookie-cutter response. […] I respond better when somebody that’s part of my medical team wants to work with me as a whole being rather than just individual parts.	P14	Q9
Actionable	They have to work together to help me as a patient, and I do have a role to play as an active patient.	P4	Q10
	I’d rather someone say, “These are some of the things that we know can help reduce pain.” Like, for example, meditation, or maybe just meeting up with a friend or making sure you have a social connection like once a week. Those may help you manage your pain. […] That’s what helps me learn, having that sort of actionable, here’s some steps to take, like read through this and then try utilizing this.	P19	Q11
Language matters	With the talk about language, use […] “biopsychosocial” immediately, that sounds very, very, very kind of woo-woo [makes hand gesture like jazz hands], […] when you start talking about biopsychosocial […] for people that are not well educated, they might not be able to pronounce it [biopsychosocial].	P17	Q12
	Ditto. I [chuckles]—honestly, I think there’s probably risk in telling someone that it can be fixed, because often it can’t, and even if it’s something that maybe normally can be fixed, everybody’s different. So living in the reality of the world and our bodies, I think aiming to reduce and cope and manage is probably the best way to present it.	P21	Q13
	I think that the prospect of a cure is, I find that amazing. I like that idea of, even in a group of 10, if one or two can reach that wall and cure their pain for even 80% of the time, or even 50% of the time, I find that really bold.	P10	Q14
Why? Information about the BPS model gives us hope	It [the pre–focus group lesson] just gave me a lot of hope to see that maybe perhaps if my mindset was changed or my approach was different that I might have better outcomes.	P16	Q15
	I was extremely excited about the thought—this for me was totally a new concept—is that pain […] was not in my head, but it was in my brain. And that sentence in the little thing we watched was huge to me. […] The whole sentence made a lot of sense, and it was relief.	P8	Q16
	I also got quite excited like [refers to another participant] when I was reading through and thinking, “Gee, there is something I can do beyond what I’ve already done.” And yes, it’s going to take work […] and I’m [going to] have to […] find out what I can do, and where I can do it, and how to do it. […] But […] it was more a case of […] I have a hope.	P20	Q17

##### Who? Everyone [All HCPs] should be sharing the BPS model

Many participants described the need for health care teams to work together and felt that information about a holistic model of pain should be shared by multiple providers (Q1). Though the general practitioner (GP) was identified as being involved in sharing pain information and being consulted for chronic pain, other participants reported that family physicians were not the primary point of contact (Q2). Practice features like frequency and duration of appointments influenced the practicality of who may be best suited to share knowledge with patients. Participants also recommended that information sharing should go beyond the health care providers and be disseminated more broadly (in and out of the clinic) (Q3). A team-based approach with consistent messaging and considerations of clinical settings may improve BPS model knowledge delivery.

##### What? Multiple tools can be used to share the knowledge

Participants suggested that multiple modalities would be suitable for supporting effective communication around the BPS model of pain. These included pamphlets and posters in health care offices, public health campaign messaging, group information sessions through a medical office, and videos like the ones shared in the pre–focus group lesson. Several participants from multiple groups reported reading medical clinic messaging, whether it be posters or handouts. A few provided specific examples when they have asked their doctor questions after reading a poster in a waiting room (Q4), and it was noted that informational posters may be pertinent to the individual’s current condition but may also provide knowledge that can be drawn upon later (Q5). Others described informational handouts prompting self-directed research, leading them to additional resources (Q6). Finally, though not discussed as prominently, several participants noted a need to change the public understanding and misconceptions through public health messaging.

##### When? HCP should introduce the BPS model earlier, and not as a last resort!

There were some divergent responses when it came to the optimal time for introduction, but there was consensus that information about the BPS model should not be introduced “as a last resort” (P11). Most participants mentioned that they would have preferred early introduction around the time when pain associated with an injury progressed from acute to chronic. Several participants also would have preferred to learn about the BPS model during the acute stage of injury or at time of diagnosis; that is, “throw it all at me” (P15).

Participants described how the timing of BPS introduction may influence how the message is received, such that an earlier introduction allows for a holistic treatment plan that supports a validating approach. Several described that sharing information about the BPS model too late could lead to invalidation when a provider that has never mentioned psychological treatment options brings it up as a last effort (Q7). In contrast, others reported that too early an introduction (Q8) or introduction during times of intense pain could similarly invalidate a person’s experience of pain. Based on these divergent views on the optimal time for BPS model introduction, the “right time” is likely to require an individualized approach, though there was consensus that the message should not be shared too late.

##### How? Work with me as a unique individual

Participants suggested elements and techniques that may be critical to improving the message/knowledge transfer. These related to tailoring the message, codesigning a plan, and an awareness of language. A latent subtheme that we developed from our codes centered on the individualization of the messaging. Participants provided examples where HCPs took time to get to know them as individuals, in contrast with times when the opposite occurred. Participants suggested going beyond a “cookie-cutter” approach and working “with me” (the client) (Q9). It was important to get to know them as a person, to understand their pain experience, and how best to work with them as an active participant in the process (Q10). When sharing pain knowledge, providers should also support patients in cocreating a plan that is “actionable” and “done with intention” (P11). Framing the message in a positive way—for example, “You might want to explore [x, y, z] to help you” (P7)—and suggesting specific things a person could do to influence their pain may improve knowledge receptivity (Q11).

Within the subtheme “how” to present the BPS model of pain to patients, participants highlighted the importance of choosing language carefully and tailoring it to the individual. Participants cautioned that using technical terms (e.g., nociception, biopsychosocial) may exclude patients from varying educational backgrounds, may sound “woo woo” (Q12), and may erode trust. In contrast, another participant (P7) noted that “they’re quite large words. That must mean something. They sound important.” Also, pertaining to language choices, there were mixed responses regarding the framing of treatment goals (e.g., to cure versus to manage), with some participants advocating for caution around framing expectations (Q13) and others wanting to “shoot [higher] for” (P8, P10) better outcomes (Q14). An individualized approach involves getting to know the client, careful selection of language and appropriate timing to suit the client, and cocreating individualized, actionable plans.

##### Why? Information about the BPS model gives us hope

A latent subtheme that we developed provides support for the importance of sharing information about the BPS model of pain to patients. When discussing participants’ reactions to the pre–focus group lesson centered on the BPS model of pain and updated pain facts, many participants reported feelings of “hope,” “[being] excited,” and “relief” (Q16, Q17). Participants reported that they wanted to learn more and that the new knowledge gave them a sense of agency over their treatment (Q18).

As described in theme 1, our participants reported that HCPs are not currently sharing this information with patients and, as such, may be omitting a potent impetus for alternative thinking about pain experience and strategies for pain management. There was a general sense of disappointment and feeling let down by HCPs for not having shared updated information regarding the current model of pain, which has potential for self-management and agency over pain.

#### Theme 6: Storytelling: Individualized evidence for the brain’s role in the manifestation of chronic pain

Our final theme was a latent thread throughout the focus group dialogue. At several points across all focus groups, participants shared personal anecdotes about self or others that provided evidence for the brain’s role in pain perception. These included some typical examples that are used in pain neuroscience education, like phantom limb and lack of awareness of an injury, along with examples of pain changing depending on fear, socioenvironmental context, and mood/emotions. Multiple participants suggested that their anticipation and fear of pain likely play a role in amplifying (and perpetuating) their experience of pain.

The anticipation of that little poke, and it was so quick, and it really wasn’t [that bad]. Like the first time I had it done, I went, “Okay, well, that was a pinch.” But then after months and months, it got worse and worse and on that day [of regular treatment], nobody could talk to me. I was so stressed. Don’t talk to me [participants laugh]. (P8)

Several others described examples when stress, emotions, and relationships triggered or worsened their pain. P13 with OA noticed stress as a factor that influences her (shoulder) pain, “It was stress that would make things worse and make things ease away.” P3 with LBP stated, “My wife would always say, ‘When you’re stressed, your back flares up.’ […] It was always in the spring that things would really get bad. And that was at the end of my school year when I was really tired and stressed.” P20, with FM and OA, noticed that her pain is influenced by social interactions: “I find it’s not so much my emotions that are causing more pain, it’s—it can be emotions, ’cause, like [named a participant] said, when certain people come around or certain tones get used.” Similarly, P5 with knee and back pain described specific relationships affecting her pain: “I know when there’s a certain person that decides to come over [chuckles] my arthritis goes nuts.”

Without prompting, several participants provided examples of contextual factors affecting pain. One of our patient partners (FG1, back pain) noted that certain sounds or changes in the environment can influence her pain: “It’s the movement of someone coming towards me and I’m reacting to that because I don’t want to fall. There are many different variables that I didn’t identify as triggers.” P21 acknowledged after a moment of reflection that the example she described when talking about emotions and pain was “a beautiful illustration” of the BPS model of pain.

I wasn’t feeling any pain at the time and I was triggered by something that rose up the emotions, right? And all of a sudden things started hurting [chuckles] that weren’t hurting before. So, yeah, it was a beautiful illustration [chuckles] of what we were about to talk about. (P21)

Other examples of evidence for the brain’s role in pain were situations where a person felt no pain despite tissue injury or the pain changed locations. These included a participant’s (P14) story about hurting themselves (though not knowing it until 2 days later): “I dropped a mirror […] on my foot in the driveway, and because it didn’t hurt, I didn’t think anything was wrong […] but I actually broke a toe.” Another participant described a loved one who should have been in excruciating cancer pain but felt no pain: “The doctors and nurses all said, ‘He should be in an enormous amount of pain, but he is expressing no sense of pain,’ but they would say, ‘The brain has a funny way of dealing with it.’” (P15). This same participant described her realization of the brain’s role in pain perception when her chronic shoulder pain disappeared when she experienced an acute hip injury.

So, I’ve been dealing with [left] shoulder pain and I’ve been doing the physio[therapy], chiro[practic], acupuncture, stretching, all the things, and I can live with it. And then I had an acute pain with my hip, left side again, and that happened and lasted for probably about six weeks, and the shoulder just became a nonissue. And then when the hip cleared up, now the shoulder’s bothering me again! And I’m, like, well that’s weird. (P15)

Finally, we note two participants observations about the brain’s role in pain as it relates to trauma. P12 with scoliosis described the start of a pain journey coinciding with a traumatic event: “It’s interesting because with my husband having Parkinson’s, he’s passed away now, but while he was dying, that’s when my pain started, so I’m—I’m guessing it’s stress related.” P15 reflected on strong emotions, like grief, presenting as physical pain.

Even if you think about grief, I mean we’ve probably all experienced the loss of someone really, really close to us, and for me, there was physical pain associated with that, and it wasn’t like my shoulder hurt […], but I had physical pain that I experienced when I was going through that. (P15)

Facilitating opportunities to listen to patient stories may provide HCPs a segue to broach the topic of the BPS model of pain, and storytelling may be an effective way to share knowledge via passive modalities (i.e., posters, pamphlets).

## Discussion

We explored participants’ knowledge of the BPS model of pain, along with preferred methods and techniques to support a sensitive introduction of the model. Based on our results, a patient-oriented approach to information sharing must begin with validation, be individualized, and involve cocreation. We suggest that system-level barriers may affect knowledge delivery and person-level barriers may affect knowledge receptivity and that both must be considered when codeveloping resources to support a patient-centered knowledge exchange. Finally, we found that a brief (15-min) introductory lesson combined with a 90-min focus group discussion about ways to introduce the BPS model resulted in significant knowledge change in our sample of people living with pain.

Gaining an understanding that pain is multifactorial (i.e., “thoughts, emotions, and experiences affect pain”) is a key concept identified by patients to support optimal pain management.^16^ Many clinical practice guidelines recommend treating individuals using a BPS approach,^6^ yet most participants in the current study reported never having learned about this model from their HCPs. Our results align with findings of a lack of uptake of the BPS model in practice settings like physiotherapy and primary care.^4,5^ Though participants in our study had some knowledge of the BPS model, our quantitative data demonstrate a knowledge gap, and many participants reported misconceptions surrounding the bidirectional nature of the relationships between pain and psychosocial factors. This was not surprising given that many HCPs similarly make one-way attributions between pain and distress but neglect to consider the reverse.^52^ An accurate depiction of the BPS model may provide a rationale for patients to seriously consider nonpharmacological options, many of which are considered first-line treatments (e.g., education, movement, cognitive behavioral therapy).^6,9,52,53^ In contrast, a lack of understanding can result in skepticism about, and lack of motivation to pursue, nonpharmacological treatments, further compounded with a fear of stigma commonly tied to mental health treatments.^53,54^ Based on our discussions with people living with pain and other studies similarly reporting lack of knowledge, there is a need for HCPs to introduce and accurately describe the BPS nature of pain to patients.

Participants described barriers that affect both delivery of and receptiveness to knowledge about the BPS model of pain. Conversations about the BPS model are not occurring, and systems-level barriers may play a role.^9,54^ Participants felt that health care appointments were too brief to facilitate a supportive introduction. Lack of time to sufficiently communicate and explore multiple biopsychosocial factors has been cited consistently by HCPs.^4,5,54^ Our participants also assumed that their providers must not be aware of the BPS model of pain. Lack of knowledge, training, and non-evidence-based beliefs among providers are ongoing concerns and are barriers to BPS model uptake in practice.^4,9,54,55^ Pain education accounts for a fraction of HCP training, despite patients with chronic pain accounting for one-third of the average Canadian family physician’s patient load^56^ and chronic pain being among the top reasons people seek medical care.^52^ Given the lack of education and misunderstood bidirectional nature of the BPS model, it is not surprising that the biomedical model is still dominant in health care.^52^ A bias toward conceptualizing pain through a biomedical lens is present at both sides of the knowledge exchange (people living with pain and HCPs) and is a barrier to knowledge delivery that further perpetuates the misconception that pain is an indicator of tissue damage or injury.^5,52^ Though the BPS model dominates in the literature and is prioritized by pain organizations worldwide, its value is undermined by a lack of uptake or attempts at late integration into pain management plans.^57^ Further, the bias may be perpetuated by so-called hidden curricula that are unconsciously shared by HCP mentors to trainees regarding the perceived lower value of psychosocial approaches while biomedical causes and treatments are commonly exhausted first.^57,58^

The biomedical bias not only serves as a barrier to knowledge delivery but also affects receptivity. Specific factors identified that may affect BPS model receptivity relate to having tissue/structural-based diagnoses and fears of psychological attributions. Public beliefs informed by a structural pathological model of pain are widespread and create a significant barrier to accepting the BPS model of pain, thereby affecting patient expectations for treatment.^59,60^ Several participants in our sample with a structural or pathological diagnosis from an authority questioned whether the BPS model applied to them. There may be a tendency for an attribution bias, such that patients who have received diagnoses may be more likely to attribute their pain experience to physical/structural causes, potentially decreasing receptivity to the BPS model. Attribution biases have been reported in samples of patients with LBP, where pain is attributed to structural factors only, and radiographic findings influence beliefs about pain severity, disability, and activity avoidance.^61,62^ When considering tools to support BPS model introduction, it may be important to explicitly state that the BPS model applies to *all* pain conditions given that reattributions of the causes of pain (i.e., from structural to psychological or multidimensional) have been linked to improved outcomes.^18,63,64^

Structure-based attributions may be due to individual biases and misinformation but also to stigma toward psychological attributions. Participants in our study expressed a fear of blame and/or being labeled as “crazy” when considering that their pain could be (in part) attributed to psychological factors. Similarly, several studies report that using psychological factors to explain the cause of pain is experienced as disbelief or a challenge to one’s integrity and delegitimizes the pain experience.^65,66^ Stigma toward psychological treatments create a significant barrier to knowledge receptivity.^53,54^ One way to lessen fears of psychological attributions is through empathetic, active validation. Our participants expressed the importance of validation when engaging in any form of knowledge exchange such that the information will not land if the client feels that they are not believed and if there is not due consideration for the impact pain has on their life. The importance of validating the pain experience (and the failure to do so) has been widely discussed in qualitative research on pain care^67,68^ and is considered a top priority by people living with pain, particularly validation that includes trust, belief of one’s experience, and respecting the knowledge they bring to the exchange.^69^ Exchanges to promote knowledge about the BPS model of pain must prioritize validation through therapeutic alliance above the content sharing.

Though participants in our study shared experiences of psychological factors influencing pain, there was a notable absence of social context in the discussions (e.g., chronic struggle,^70^ sociodemographic status, gender, employment, race and ethnicity). We note that our introductory lesson and script provided examples of social factors and were framed to discuss the complex interactions within the BPS mo nonetheless, social context beyond the personal or individual levels (e.g., occupation and relationship triggers) did not surface in our groups. The lack of discourse on social context and its impact on pain is evidenced in physiotherapy and broadly in the literature where the conceptualization of the BPS model largely focuses on physiological and psychological factors^71,72^ and on person-level social contexts ignoring social structures.^70^ The absence of social context in our discussions may speak to participants belonging to dominant groups within broader social structures or to a lack of awareness and understanding of how social structures operating beyond the person level have potential to influence a person’s pain experience.^70,71^

Implementation strategies for BPS model introduction will require careful consideration of barriers and facilitators to knowledge delivery and receptivity and be informed by patient views on preferred modalities, techniques, and timing. We extracted several subthemes to help guide tool and resource codevelopment for BPS model introduction. Participants reported that all HCPs should be sharing this information. A need for consistent messaging about pain by all HCPs aligns with calls for multidisciplinary pain management,^7^ and a team-based and holistic approach to pain management is a priority for both HCPs and people living with pain.^69^ Participants in our study were open to multiple modalities for passive knowledge sharing, including posters, pamphlets, videos, and public messaging campaigns. Patient health leaflets/posters in waiting rooms have high (94%) readership and may encourage discussions with providers,^73^ though knowledge sharing via digital screens tends to be more effective.^74^

Though passive modalities appealed to our groups to support learning at a self-selected pace, participants also recommended patient-centered ways of sharing the knowledge that went beyond validation. These included working *with* them as individuals and sharing the knowledge with an action plan. Similarly, Slater et al. reported care priorities for people living with pain related to a holistic, tailored approach and ensuring a genuine partnership in their pain care.^69^ A lack of partnership has resulted in patient–provider interactions that have mismatched goals (e.g., wanting to be understood versus wanting to treat).^61^ Knowledge sharing that considers different beliefs, fear of stigma, and validation may best involve a two-way exchange where both provider and patient share beliefs about how they conceptualize pain. Cocreating a narrative about pain may provide a foundation for a treatment plan that incorporates a variety of BPS-informed approaches (i.e., both passive and active management strategies), thereby dually respecting the patient’s autonomy while adhering to clinical guidelines.^61,69^

Though sharing knowledge may challenge patient beliefs, our participants reported that people living with pain deserve to know early in their journey that pain is multifactorial and that by influencing some of the factors a person can influence their pain experience. Information about the psychosocial influences of pain is commonly introduced late in a pain journey, resulting in missed opportunities for evidence-based self-management practices that can improve pain and quality of life.^57^ If there a “sweet spot” for BPS model introduction, it likely involves an individualized approach, as described above, developing enough rapport to provide validation and work with the patient to then allow space for the introduction. Given that ignoring psychosocial yellow flags is a risk factor for developing pain chronicity and disability,^75^ pain communication would best involve an introduction to the BPS model during the acute phase of an injury or postsurgical period or in the early stages of disease.

The most highly valued priorities by patients include learning from them, inviting their expressions of pain, and listening and acknowledging their pain stories to foster shared understanding.^66,69^ Our results similarly suggest that exploring personal narratives with curiosity may provide a segue to a sensitive introduction of the BPS model between a client and provider. To facilitate storytelling, HCPs will need to provide opportunities to listen to the patient’s pain journey and experiences, which aligns with our participants’ desire to be validated and treated as individuals. Providers may ask prompting questions about whether pain changes over time and place (e.g., using techniques like motivational interviewing). Storytelling through posters and pamphlets may also serve as a technique to support BPS model introduction, given that narrative methods for health communication show higher appeal and effectiveness compared to didactic methods.^76^ Our findings are aligned with current directions in pain science education, which are evolving to incorporate techniques like storytelling and active learning.^18^

Strengths of our study include a heterogeneous sample of participants with mixed pain conditions and varied employment status and representing a range of ages. We collected clinical and psychological data to provide sample characteristics for several common clinical features associated with chronic pain to better inform decisions around the transferability of the results. The focus group method for data collection resulted in rich discussions, where participants demonstrated balanced sharing, piggybacked off each other’s ideas, and included both agreement and collegial disagreement. We employed a reflexive qualitative framework that included time for data familiarization, coding by investigators with diverse experiences (i.e., student, clinician, researcher), and different types of engagement with a BPS-informed model of health and/or pain (i.e., lived experience, pain practitioner). Finally, our patient partners provided guidance and shared decision making throughout the research process.

Limitations of our study include limited gender and educational diversity, with only three participants identifying as men, and participants were well-educated, with most having completed a university degree, though we note that our participants represented a range of household incomes. Further, though we gathered basic sociodemographic data (e.g., gender, income, education, employment, relationship status), we acknowledge that additional data on ethnicity, social support, and access to health care would have provided more comprehensive sample characterization for social determinants commonly associated with pain.^77–79^ In particular, the absence of data to determine proportion of underrepresented racial and ethnic groups in our sample limits considerations of transferability. Thus, our results represent the views of this unique British Columbian sample of people living with pain and results may not be as applicable in other contexts. We note, however, that many of our findings are consistent with qualitative and quantitative findings in pain populations, and our results may be used to guide tool codevelopment for further testing on larger, diverse groups of people living with pain in a variety of contexts. Finally, we note that the observed changes in pain knowledge are limited by the lack of a control group.

### Implications

Our findings demonstrate that there is a need for sharing knowledge about the BPS model of pain with patients. Closing the knowledge gap will require system-level changes to health professional education, some of which are underway at a national level guided by Canada’s Pain Task Force.^80^ These include enhanced pre- and postlicensure training on treating pain through a BPS lens, exploring competency frameworks for health professionals, reflecting on the hidden curriculum and culture within respective disciplines, and championing BPS-informed practice across disciplines. To support BPS introduction in practice, we propose the codevelopment of resources for HCPs to support empathetic validation and explore pain experiences with compassionate curiosity. Provider resources can be combined with patient-facing resources to engage patients at their own pace and allow in-person time to be focused on listening and validation. Given that many participants used storytelling to share experiences illustrating the brain’s role in the pain experience, our results provide potential techniques to spark a dialogue. Moving forward, to inform the codevelopment of tools to support BPS model introduction with stakeholders, it will be important to better characterize sociodemographic features of stakeholder groups and to explore the impact of various social dimensions on a sensitive introduction to the BPS model of pain.

## Conclusion

We examined the current state of knowledge about the BPS model of pain in people living with pain, along with patient-centered ways to best introduce the concept that pain is a complex, multifactorial experience. This study demonstrates the need for sharing knowledge about the BPS model of pain, based on the low pain knowledge at baseline, the strong desire for nonpharmacological options for pain management, and the sense of hope expressed by our participants following study exchanges. The themes extracted from our participants may be used to guide tool development to support a patient-centered introduction to the BPS model of pain in practice.

## Supplementary Material

Supplementary File v2 CLEAN.docx

## Data deposition

Due to the descriptive stories shared by participants, it is not possible to share full data set, which would compromise confidentiality, but the data sets for baseline and post–focus group questionnaires are available in the Open Science Framework (https://doi.org/10.17605/OSF.IO/6RKEQ).
