# Supplementary material for: Let’s talk about pain: A mixed methods exploration of patients’ pain knowledge and preferred ways to introduce the biopsychosocial model of pain
Source: Can J Pain. 2026 May 11;10(1):2650304. doi: 10.1080/24740527.2026.2650304 (PMC13166251; doi:10.1080/24740527.2026.2650304)
Supplement: Supplementary File v2 CLEAN.docx [file UCJP_A_2650304_SM7281.docx]

**Supplementary Digital Content**

## **Table of Contents Page**

[Supplementary Content 1: Focus Group Guiding Questions](#_Supplementary_Content_1:) S2

[Supplementary Content 2: Patient Partners, Full description](#_Supplementary_Content_2:) S4

[Supplementary Content 3: Pre-focus group lesson content](#_Supplementary_Content_3:) S5

## **Supplementary Content 1:**

## **Box 1. Focus Group Guiding Questions**

| **Introduction**   - Facilitator and researchers introduce themselves. Provide notification of taping and ground rules for group discussion. Ice breaker introduction of participants, followed by study objectives.   **Part 1: Reflections from lesson**   - Summary slide of lesson is displayed for reference. Prompts by facilitator:   - Have you heard this <BPS model of pain> before? Was this new information?   - Did the information in the lesson align with what you’ve been told previously?   - What stood out to you? Key take aways?   - Were you surprised by any information (good or bad)?   - Facilitator then reads a statement about pain (similar to the example presented in the lesson) and asks group to consider how it makes them feel.   *“A normal stimulus, such as your brain detecting where your arm is in space may be perceived by the brain as painful, despite there being no tissue damage. In someone without chronic/persistent pain, the brain may interpret the signal as, “ok, my arm just raised above my head.” In a person with pain, they may have an expectation that raising an arm usually triggers pain, and this can then amplify the experience of pain. In other words, pain involves perception at the level of the brain. Some researchers describe pain as “an opinion” or that the brain “decides” what will be experienced as pain.”*  **Part 2: How to introduce the BPS model of pain**   - Facilitator asks the group about ways to communicate information about the complex nature of pain in a brief medical appointment.   - Do you have suggestions about how a practitioner could introduce this information?   - Where is the best opportunity to learn more about the neurophysiology of pain? E.g., Who or what type of provider? Is there an optimal time or place?   - What language or introduction may allow you to be receptive to hearing a provider recommend a psychologically based treatment option?   - What language might make you less receptive to this message?   - If you were telling another person in pain about the BPS model, how would you broach the subject?   - Would you be interested in a pamphlet? A video? Digital platforms? Or is individual discussion preferred? Are there other ways that we haven’t thought of?   - What characteristics would allow a person in pain to be receptive to this message? |
| --- |

## **Supplementary Content 2: Patient Partners**

Our patient partners included a man (university administrator) and a woman (registered nurse) who had experienced or had ongoing persistent back pain that resulted in time away from work. They were independently referred to a Work Safe counselling program with a pain psychologist [A4] (former patients, not under A4’s care at time of the study) through which they gained an in-depth understanding of the BPS model of pain. Neither had served as patient partners previously. Before study design and planning began, the primary investigator [A1] met with partners to discuss their goals, questions, roles, and emphasized the value and perspectives of diverse study team members and the plan for shared decision making. All team members actively participated in planning meetings, including document preparation (*e.g*., script writing, survey creation, recruitment posts, pre-lesson planning), active study period (*e.g*., attendance at focus groups), and review of results. We invited partners to participate in all analyses (transcript review, coding, theme review), but they self-selected out of the coding meetings and chose to contribute to the final review of the themes. All study documents were collaboratively reviewed using online sharing platforms and meetings were held virtually.

## **Supplementary Content 3: Pre-focus group lesson content**

Structured to be a 15 minute lesson titled “What is Pain?”, presented using Canva online platform. Participants clicked on an arrow, self-paced through the lesson slides. Below are summaries for the content on each slide (numbered 1 through 11).

1. **Overview** guiding the user how to move through the presentation using arrows and how to play videos. We recommend if you have questions, to write them down so that we can discuss them in the focus groups. If some of the information makes you feel uncomfortable, please take note of how it makes you feel and bring this information to the focus group.
2. **Pain**: A brief description of acute pain (e.g., you stub your toe) and persistent pain (e.g., sustained pain beyond the usual healing time).
3. **How does pain work?** A flow diagram depicting nociception (using lay language) and illustrating an acute example of a stubbed toe. In brief the slide depicts a “danger” message sent via the spinal cord to the brain. And there is a summary to explain that “no matter where we feel pain in the body, pain is processed in the brain and the brain decides if we feel pain”. The figure also notes that pain is like an alarm signal that serves to protect the body from harm. (information on the slide reformatted from Ontario Pain Network, 2020).
4. **What about when pain becomes persistent?** Embedded within the slide is the [Tame the Beast](https://www.youtube.com/watch?v=ikUzvSph7Z4) (5 minute) video by tamethebeast.org.
5. How does persistent pain work? (a follow up to slide 3 that demonstrated acute pain) In persistent pain, the nervous system gets more sensitive, like a fire alarm that goes off for no reason or sounds louder. The pain alarm is trying to protect you from future dangers by changing brain pathways that regulate emotions, memory, movement, stress, and so on.
6. **Five Key Points About Pain** (Ontario Pain Network, 2020). These included:
   1. 1 in 5 Canadians experience persistent pain.
   2. Your brain determines what you experience as pain.
   3. All pain is real, even if you cannot see the cause.
   4. Pain is an experience that is a result of many different factors.
   5. Pain is not an accurate reflection of tissue damage.
   6. By managing these factors, we can make a difference to our pain.
7. **Pain is a Biopsychosocial Phenomenon**: Meaning that pain is influenced by our biology (physiology), our thoughts and emotions (e.g., psychology), and our relationships/interactions with others (sociology).
8. **Considering the biopsychosocial model of pain, many things can impact your pain**. What contributes to your pain? Listed were several examples like: things you say, things you believe, places you go, etc. (Content from Ontario Pain Network, 2020).
9. To recap (with some redundancy noted) we showed another 5-minute video, “[Understanding Pain in less than 5 minutes, and what to do about it”](https://www.youtube.com/watch?v=C_3phB93rvI) (often referred to as the “Brainman” video created by Hunter Integrated Pain Service and collaborators).
10. **Summary**:
    1. Pain is a very real experience. You feel it and it hurts! The sensation are real.
    2. Pain is not an accurate reflection of tissue damage, as most tissue heals within about 3 to 6 months after injury.
    3. With persistent pain, the nervous system becomes more sensitive to signals coming from the body and interprets them as threats.
    4. So, while an injury can often result in acute pain, persistent pain is learned. The brain has learned to create pain in the absence of tissue damage.
    5. With persistent pain, the nervous system becomes more sensitive to neutral signals coming from the body and interprets them as threats which results in pain, rather than interpreting them as normal cues regarding (for example) body position.
    6. Pain is influenced by our thoughts, emotions, and interactions with others. It is a Biopsychosocial phenomena.
    7. Just as our brains can learn pain, our brains are plastic and malleable, so we can work towards unlearning our pain by considering biological, psychological, and social factors that influence our pain.
